# Supplementary material for: Divergent effects of azithromycin on purple corn (Zea mays L.) cultivation: Impact on biomass and antioxidant compounds
Source: PLoS One. 2024 Aug 22;19(8):e0307548. doi: 10.1371/journal.pone.0307548 (PMC11340972; doi:10.1371/journal.pone.0307548)
Supplement: S1 Table — (PDF) [file pone.0307548.s001.pdf]

SUPPORT INFORMATION

Table S1. Behavior of total phenolic compounds, anthocyanins, and antioxidant capacity in the husk of purple corn exposed to azithromycin

**Cocentration de AZM µg/L**	Polyphenols *mg GAE/g dw*		Anthocyanins *mg EC3G/ g dw*		IC_{50}		ORAC *µmol TE/g dw*	
	$\bar{X}$	SD	$\bar{X}$	SD	$\bar{X}$	SD	$\bar{X}$	SD
0	29.09a	±2.76	13.895a	±5.84	2.41b	±0.79	1207.86b	±98.96
1	48.03b	±3.93	21.030b	±41.46	2.18a,b	±0.19	1099.40a	±87.84
10	61.74d	±3.48	29.532b	±53.24	1.50a	±0.39	2592.15c	±430.94
100	34.95c	±2.56	13.947a	±45.44	1.76a	±0.36	1223.53b	±53.21

$\bar{X}$ represents the mean (n=3). SD represents the standard deviation. The total polyphenol content is expressed as mg of gallic acid equivalent per gram of dry weight (mg GAE/g dw). The anthocyanin content is expressed as mg of cyanidin-3-glucoside per gram of dry weight (mg EC3G/ g dw). The IC_{50} value is mg of extract per mL of DPPH solution. The ORAC value is the micromoles of Trolox equivalent per gram of dry weight (µmol TE/g dw). Different letters indicate significant differences for each response variable ($p < 0.05$).
