# Supplementary material for: Divergent effects of azithromycin on purple corn (Zea mays L.) cultivation: Impact on biomass and antioxidant compounds
Source: PLoS One. 2024 Aug 22;19(8):e0307548. doi: 10.1371/journal.pone.0307548 (PMC11340972; doi:10.1371/journal.pone.0307548)
Supplement: Attachment — Submitted filename: PONE-D-24-03135_reviewer commemts.pdf [file pone.0307548.s002.pdf]

PLOS ONE

Divergent effects of Azithromycin on purple corn (Zea mays L.) cultivation: Impact on biomass and antioxidant compounds

--Manuscript Draft--

**Manuscript Number:**	PONE-D-24-03135
**Article Type:**	Research Article
**Full Title:**	Divergent effects of Azithromycin on purple corn (Zea mays L.) cultivation: Impact on biomass and antioxidant compounds
**Short Title:**	Azithromycin effects on purple corn: Impact on biomass, antioxidants diverge
**Corresponding Author:**	Franz Vilca<br>Universidad nacional de Moquegua<br>Moquegua, PERU
**Keywords:**	Homesis, toxicity, antibiotics, soil
**Abstract:**	The present study assessed the impact of using irrigation water contaminated with Azithromycin (AZM) residues on the biomass and antioxidant compounds of purple corn; for this purpose, the plants were cultivated under ambient conditions, and the substrate used consisted of soil free from AZM residues, mixed with compost in a ratio of 1:1 (v/v). The experiment was completely randomized with four replications, with treatments of 0, 1, 10, and 100 µg/L of AZM. The results indicate that the presence of AZM in irrigation water at doses of 1 and 10 µg/L increases the weight of dry aboveground biomass, while at an amount of 100 µg/L, it decreases. Likewise, this study reveals that by increasing the concentration of AZM from 1 to 10 µg/L, total polyphenols and monomeric anthocyanins double, in contrast, with an increase to 100 µg/L, these decrease by 44 and 53%, respectively. It has been demonstrated that purple corn exposed to the antibiotic AZM at low doses has a notable antioxidant function in terms of DPPH and ORAC. The content of flavonols, phenolic acids, and flavanols increases by 57, 28, and 83%, respectively, when the AZM concentration is from 1 to 10 µg/L. However, with an increase to 100 µg/L, these compounds decrease by 17, 40, and 42%, respectively. On the other hand, plant height, root length, and dry weight of root biomass are not significantly affected by the presence of AZM in irrigation water
**Order of Authors:**	Franz Vilca
	Yoselin Mamani Ramos
	Nils Huaman Castilla
	Elvis Jack Colque Ayma
	Noemi Mamani Condori
	Clara Nely Campos Quiróz
**Additional Information:**	
**Question**	**Response**
**Financial Disclosure**<br><br>Enter a financial disclosure statement that describes the sources of funding for the work included in this submission. Review the submission guidelines for detailed requirements. View published research articles from *PLOS ONE* for specific examples.	Yes

Powered by Editorial Manager® and ProduXion Manager® from Aries Systems Corporation

This statement is required for submission and **will appear in the published article** if the submission is accepted. Please make sure it is accurate.<br><br>**Funded studies**<br>Enter a statement with the following details:<br>• Initials of the authors who received each award<br>• Grant numbers awarded to each author<br>• The full name of each funder<br>• URL of each funder website<br>• Did the sponsors or funders play any role in the study design, data collection and analysis, decision to publish, or preparation of the manuscript?<br><br>Did you receive funding for this work?	
Please add funding details.<br>as follow-up to "**Financial Disclosure**<br><br>Enter a financial disclosure statement that describes the sources of funding for the work included in this submission. Review the submission guidelines for detailed requirements. View published research articles from *PLOS ONE* for specific examples.<br><br>This statement is required for submission and **will appear in the published article** if the submission is accepted. Please make sure it is accurate.<br><br>**Funded studies**<br>Enter a statement with the following details:<br>• Initials of the authors who received each award<br>• Grant numbers awarded to each author<br>• The full name of each funder<br>• URL of each funder website<br>• Did the sponsors or funders play any role in the study design, data collection and analysis, decision to publish, or preparation of the manuscript?<br><br>Did you receive funding for this work?"	Resolución de Comisión Organizadora N° 0310–2020—UNAM and Resolución de Comisión Organizadora N° 059–2021—UNAM

Please select the country of your main research funder (please select carefully as in some cases this is used in fee calculation).<br>as follow-up to "**Financial Disclosure**<br><br>Enter a financial disclosure statement that describes the sources of funding for the work included in this submission. Review the submission guidelines for detailed requirements. View published research articles from *PLOS ONE* for specific examples.<br><br>This statement is required for submission and **will appear in the published article** if the submission is accepted. Please make sure it is accurate.<br><br>**Funded studies**<br>Enter a statement with the following details:<br>• Initials of the authors who received each award<br>• Grant numbers awarded to each author<br>• The full name of each funder<br>• URL of each funder website<br>• Did the sponsors or funders play any role in the study design, data collection and analysis, decision to publish, or preparation of the manuscript?<br><br>Did you receive funding for this work?"	PERU - PE
**Competing Interests**<br><br>Use the instructions below to enter a competing interest statement for this submission. On behalf of all authors, disclose any competing interests that could be perceived to bias this work—acknowledging all financial support and any other relevant financial or non-financial competing interests.<br><br>This statement is required for submission and **will appear in the published article** if the submission is accepted. Please make sure it is accurate and that any funding	NO authors have competing interests

sources listed in your Funding Information later in the submission form are also declared in your Financial Disclosure statement.<br><br>View published research articles from *PLOS ONE* for specific examples.<br><br>**NO authors have competing interests**<br><br>Enter: *The authors have declared that no competing interests exist.*<br><br>**Authors with competing interests**<br><br>Enter competing interest details beginning with this statement:<br><br>*I have read the journal's policy and the authors of this manuscript have the following competing interests: [insert competing interests here]*<br><br>* typeset	
**Ethics Statement**<br><br>Enter an ethics statement for this submission. This statement is required if the study involved:<br><br>• Human participants<br>• Human specimens or tissue<br>• Vertebrate animals or cephalopods<br>• Vertebrate embryos or tissues<br>• Field research<br><br>Write "N/A" if the submission does not require an ethics statement.<br><br>General guidance is provided below. Consult the submission guidelines for detailed instructions. **Make sure that all information entered here is included in the Methods section of the manuscript.**	N/A

**Format for specific study types**<br><br>**Human Subject Research (involving human participants and/or tissue)**<br>• Give the name of the institutional review board or ethics committee that approved the study<br>• Include the approval number and/or a statement indicating approval of this research<br>• Indicate the form of consent obtained (written/oral) or the reason that consent was not obtained (e.g. the data were analyzed anonymously)<br><br>**Animal Research (involving vertebrate animals, embryos or tissues)**<br>• Provide the name of the Institutional Animal Care and Use Committee (IACUC) or other relevant ethics board that reviewed the study protocol, and indicate whether they approved this research or granted a formal waiver of ethical approval<br>• Include an approval number if one was obtained<br>• If the study involved *non-human primates*, add *additional details* about animal welfare and steps taken to ameliorate suffering<br>• If anesthesia, euthanasia, or any kind of animal sacrifice is part of the study, include briefly which substances and/or methods were applied<br><br>**Field Research**<br><br>Include the following details if this study involves the collection of plant, animal, or other materials from a natural setting:<br>• Field permit number<br>• Name of the institution or relevant body that granted permission	
**Data Availability**<br><br>Authors are required to make all data underlying the findings described fully available, without restriction, and from the time of publication. PLOS allows rare exceptions to address legal and ethical concerns. See the PLOS Data Policy and FAQ for detailed information.	Yes - all data are fully available without restriction

A Data Availability Statement describing where the data can be found is required at submission. Your answers to this question constitute the Data Availability Statement and **will be published in the article**, if accepted.<br><br>**Important:** Stating 'data available on request from the author' is not sufficient. If your data are only available upon request, select 'No' for the first question and explain your exceptional situation in the text box.<br><br>Do the authors confirm that all data underlying the findings described in their manuscript are fully available without restriction?	
**Describe where the data may be found in full sentences. If you are copying our sample text, replace any instances of XXX with the appropriate details.**<br><br>• If the data are **held or will be held in a public repository**, include URLs, accession numbers or DOIs. If this information will only be available after acceptance, indicate this by ticking the box below. For example: *All XXX files are available from the XXX database (accession number(s) XXX, XXX.).*<br>• If the data are all contained **within the manuscript and/or Supporting Information files**, enter the following: *All relevant data are within the manuscript and its Supporting Information files.*<br>• If neither of these applies but you are able to provide **details of access elsewhere**, with or without limitations, please do so. For example:<br><br>*Data cannot be shared publicly because of [XXX]. Data are available from the XXX Institutional Data Access / Ethics Committee (contact via XXX) for researchers who meet the criteria for access to confidential data.*<br><br>*The data underlying the results presented in the study are available from (include the name of the third party*	No

and contact information or URL).<br>• This text is appropriate if the data are owned by a third party and authors do not have permission to share the data.<br><br>* typeset	
Additional data availability information:	

Powered by Editorial Manager® and ProduXion Manager® from Aries Systems Corporation

Click here to access/download;Manuscript;Divergent Effects of Azithromycin final_NH.docx

Divergent effects of Azithromycin on purple corn (*Zea mays L.*) cultivation: Impact on biomass and antioxidant compounds

Yoselin Mamani Ramos[1,2], Nils Huaman Castilla[3,4] Elvis Jack Colque Ayma[2], Noemi Mamani Condori[1,2], Clara Nely Campos Quiróz[2] and Franz Zirena Vilca[1,2¶].

[1]Escuela profesional de Ingeniería Ambiental de la Universidad Nacional de Moquegua, Urb Ciudad Jardín-Pacocha-Ilo- Perú.

[2]Laboratorio de Contaminantes Orgánicos y Ambiente del IINDEP de la Universidad Nacional de Moquegua, Urb Ciudad Jardín-Pacocha-Ilo- Perú.

[3]Escuela profesional de Ingeniería Agroindustrial, Universidad Nacional de Moquegua, Prolongación Calle Ancash S/N, Moquegua 18001, Perú.
.
[4]Laboratorio de Tecnologías Sustentables para la Extracción de Compuestos de Alto Valor, Instituto de Investigación para el Desarrollo del Perú, Universidad Nacional de Moquegua, Prolongación Calle Ancash S/N, Moquegua 18001, Perú.

* Corresponding author

E-mail: fzirenav@unam.edu.pe (FZV)

Abstract

The present study assessed the impact of using irrigation water contaminated with Azithromycin (AZM) residues on the biomass and antioxidant compounds of purple corn; for this purpose, the plants were cultivated under ambient conditions, and the substrate used consisted of soil free from AZM residues, mixed with compost in a ratio of 1:1 (v/v). The experiment was completely randomized with four replications, with treatments of 0, 1, 10, and 100 μg/L of AZM. The results indicate that the presence of AZM in irrigation water at doses of 1 and 10 μg/L increases the weight of dry aboveground biomass, while at an amount of 100 μg/L, it decreases. Likewise, this study reveals that by increasing the concentration of AZM from 1 to 10 µg/L, total polyphenols and monomeric anthocyanins double, in contrast, with an increase to 100 µg/L, these decrease by 44 and 53%, respectively. It has been demonstrated that purple corn exposed to the antibiotic AZM at low doses has a notable antioxidant function in terms of DPPH and ORAC. The content of flavonols, phenolic acids, and flavanols increases by 57, 28, and 83%, respectively, when the AZM concentration is from 1 to 10 µg/L. However, with an increase to 100 µg/L, these compounds decrease by 17, 40, and 42%, respectively. On the other hand, plant height, root length, and dry weight of root biomass are not significantly affected by the presence of AZM in irrigation water.

Introduction

Purple corn (*Zea Mays L.*) is a distinct variety of maize emblematic of Peru, whose production reached 24.5 thousand tons in 2020 (1). This product has gained considerable attention recently due to its rich polyphenol and anthocyanin content and potential health benefits (2). In particular, the corn crown has high concentrations of polyphenols (225 mg GAE/100 g dw) and anthocyanins (2600-3800 mg/100 g dw) (3, 4), which have

demonstrated their effectiveness in the treatment of diseases related to oxidative stress (5) (6). For example, phenolic acids, flavonols, and stilbenes are polyphenols used to treat conditions like hypertension, intestinal inflammation, and cancer (7, 8). On the other hand, anthocyanins like cyanidin and delphinidin can be used to treat type 2 diabetes (9). Thus, this corn variety is a bioactive compound source and a valuable ingredient in the food and beverage industry.

Polyphenols have phenolic and hydroxyl groups in their chemical structure, whose molecular weights can vary between 160 to 30 000 Da (10, 11). These compounds are synthesized through the phenylpropanoid metabolic pathway as a protective response to stressors like temperature fluctuations, humidity levels, exposure to ultraviolet radiation, and exposure to chemical compounds such as pesticides and antibiotics (12, 13, 14). In this sense, antibiotics and pharmaceutical products can disrupt plant development and impact the synthesis of polyphenols.

Antibiotics and pharmaceuticals are used to enhance public health. Nonetheless, these substances are not fully metabolized, leading to the presence of their residues in surface and sewage waters (15, 16). For example, (17) identified the presence of elevated concentrations of antibiotic residues in surface waters, such as Sulfamethoxazole, Trimethoprim, Azithromycin (AZM), Clarithromycin, and Ciprofloxacin (CPF). Thus, these effluents are employed in the agricultural sector to irrigate various plants, and their absorption could represent a potential health risk to consumers (18). Explain How AZM will be the residue in water

Antibiotics can be adsorbed and distributed among plant tissues like roots, stems, leaves, and fruits (19, 20). For example, when AZM (23.6 ng/L) and CPF (15.6 ng/L) are present in irrigation water, plants can absorb between 5 and 15% of these compounds (21). Although the presence of antibiotics in the plant could affect its normal development, these compounds have the potential to stimulate an increased production of

polyphenols. For example, (12) demonstrated in quinoa grains that controlled exposure to CPF residues in irrigation water (100 µg/L) allowed an increase in the polyphenol content by 52% compared to the control treatment. In contrast, under these considerations, morphological characteristics such as roots, leaves, panicles, and grain were not affected; however, different plants possess variable vulnerability to other antibiotics, even inducing a hormetic response. This study explores the influence of AZM residues in irrigation water on the impact of biomass and antioxidant compounds in purple corn.

Materials and methods

The experiment was conducted under ambient conditions, using polyethylene pots with dimensions of 24 X 31.5 X 28 cm for width, height, and base, respectively, filled with 18 kg of the substrate (soil and compost) up to a height of 27 cm. Subsequently, after the plants completed their physiological cycle, the length and dry weight of the root biomass, plant height, dry weight of the aboveground biomass, and dry weight of the cob were analyzed. Total phenolic compounds and anthocyanin concentrations in the corn cob were also examined.

The planting was carried out according to the procedure proposed by (22) with minimal adjustments based on the observations of (23). In the first phase, the seeds underwent surface disinfection using a 2.5% sodium hypochlorite solution with an exposure time of 5 minutes. Subsequently, rinses with distilled water were carried out, and the seeds were placed in 18-litre pots containing a substrate mixture of soil and compost in a 1:1 ratio (v/v). Initially, each bank accommodated four sources, and after the emergence of seedlings, the most vigorous plant in each pot was selected, resulting in a single plant per container. The cultivation occurred under ambient conditions, and the plants received initial irrigation with distilled water.

The AZM stock solution was prepared in distilled water using analytical-grade AZM. This stock solution was subsequently used to create different test concentrations. The plants were irrigated with concentrations of 0 (control with distilled water) and 1, 10, and 100 μg/L of AZM, taking as a reference a previously documented range of occurrence of this antibiotic in surface waters (17). The irrigation of the plants was carried out two or three times per week, using between 1 and 2 liters of water on each occasion, aiming to maintain a substrate field capacity of 70%. Sixteen pots were arranged and distributed across four treatments, including a control (without antibiotic) and concentrations of 1, 10, and 100 μg/L of AZM, with four replications for each treatment.

Evaluation of the dry biomass of the plant

To assess the height and weight of the dry aboveground biomass, dry weight of the cob, root length, and dry weight of the root biomass, the method was proposed by (23). For this, the harvest of the plants took place at stage R6 (physiological maturity), and they were divided into roots, leaves, and cobs.

Plastic was used to secure the aboveground and root parts of the plant.
=stem length??
Subsequently, the root length and plant height were measured. Next, the plant was
How to separate?
separated into leaves and stems. Following this, drying was carried out at 60 °C for 48 hours, and then the plant material was weighed on an analytical balance. This exact procedure was conducted to determine the weight of the dry root biomass.

Extraction of phenolic compounds

The samples were ground to obtain a fine powder (approximately 500 μm), and subsequently, the sample's moisture content was determined to extract phenolic compounds.

The extraction of phenolic compounds was carried out using the method reported by (24) with slight modifications based on what was written by (25). To extract phenolic

compounds, 1g of dry powder sample was mixed with 20 mL of 60% acetone.
%v/v???
(acetone/water 60:40, %/%). The mixture was then stirred (350 rpm for 30 minutes at 35 °C). Subsequently, the homogenized mixture was centrifuged at 4000 rpm for 5 minutes, and the supernatant was recovered. A second centrifugation at 4000 rpm for 5 minutes was performed on the residue, and the second supernatant was recovered. Both
how to be sure that water also remove ?, no freezedry?
supernatants were combined and concentrated under vacuum at 40°C until dryness. The
or final volume to 10 mL???
phenolic compounds were then reconstituted in 10 mL of 60% acetone. The final aqueous extract obtained was stored in amber containers and kept at a temperature of -20°C until further analysis.

Quantification of Total Phenolic Compounds

The concentration of total phenolic compounds was determined from the crude
?? or rewrite
extract using the method proposed by (26), cited by (27). In brief, 0.5 mL of corn extract was diluted with 3.75 mL of distilled water in a flask. 0.25 mL of Folin-Ciocalteu reagent (1 N) was added to each sample. After 5 minutes of reaction, 0.5 mL of a 10% aqueous solution of sodium carbonate (Na_2CO_3) was added. The mixture was then allowed to stand at room temperature for one hour until the characteristic blue color developed. Absorbance was measured at 765 nm against a blank containing distilled water instead of the sample using a spectrophotometer. The total phenolic compound content was determined through a calibration curve prepared with gallic acid at different concentrations ranging from 10 to 90 mg/L, with a high coefficient of determination (R2) of 0.9988, where the calibration curve is $y = 0.009x + 0.00174$ The results were expressed as milligrams of gallic acid equivalents (GAE) per gram of the sample's dry weight (DW).

$$PFT\left(\frac{mg\ Gallic\ acid}{litre}\right) = \frac{(Abs\ sample - B)}{A} \times FD \qquad (1)$$

Where Abs. The sample is the absorbance of the sample, A Slope, B Intercept, and FD dilution factor. Later, this value was corrected to mgGAE/gdw, considering values obtained during the moisture determination.

Total, Anthocyanin Content (TAC)

The total anthocyanin content was determined using the differential pH method (28), with some modifications reported (29). The entire anthocyanin content was also determined from the crude extract. Before TAC determination, two solutions were prepared, one for pH 1.0 using potassium chloride buffer (0.025 M, 0.0465 g of KCl in 25 mL of distilled water, acidified with HCl), and the other for pH 4.5 using sodium acetate buffer (0.4 M, 1.3608 g of CH_3CO_2Na in 25 mL of distilled water, acidified with HCl). Subsequently, the protocol for Total Anthocyanins was followed, which involved 20 µL of extract in 80 µL of pH buffer and 20 µL of section in 80 µL of pH 4.5 buffer. Each sample's absorbance was measured at 520 nm and 700 nm. TAC was calculated using the following equation and is expressed as milligrams of cyanidin-3-glucoside (C3G) per gram of dry weight (dw) of the sample

??

$$TAC\left(\frac{mg\ EC3G}{L}\right) = \frac{A \times MW \ \times FD \times 1000}{eL} \times \frac{V}{gbh} \times \frac{gbh}{gbs} \qquad (2)$$

Where: TAC Is the concentration of total anthocyanins (mg EC3G/g bs); A It is the absorbance and is calculated as A = (A520 – A700) pH 1.0 - (A520 – A700) pH 4.5); e is the molar absorptivity of cyanidin-3-glucoside (26900 L/ (cm * mol)); L is the cell path length (1 cm); MW is the molecular weight of cyanidin-3-glucoside (449.2 g/mol); FD is the dilution factor. Subsequently, this value was corrected to mgEC3G/gdw, considering values obtained during the moisture determination.

Antioxidant capacity by DPPH

The antioxidant capacity of the extracts was evaluated using the DPPH (2,2-diphenyl-1-picrylhydrazyl) radical scavenging method (30, 12). The test involved mixing

How to prepare the solution in different conc. of extracts ?

0.1 mL of extract with 3.9 ml of a DPPH solution (0.1 mM). The resulting combination was left at room temperature in the dark for 30 minutes. After this period, the reduction of DPPH was measured by absorption at 517 nm using a UV spectrometer (UV 1240, Shimadzu, Kyoto, Japan). The determination of IC50, representing the concentration of extract needed to inhibit the absorption of DPPH radicals by 50%, was carried out based on these data. It is important to note that this parameter indicates the antioxidant efficacy of the extracts, providing valuable information about their ability to neutralize free radicals.

Antioxidant capacity by Oxygen Radical Absorbance Capacity (ORAC)

The antioxidant activity of the extracts was determined according to the methodology proposed by (31) and (12). The ORAC analyses were performed on a 96-well microplate fluorometer (Ascent FL Fluoroscan, Labsystem, Finland). To generate peroxyl radicals, 2,2'-azobis(2-amidinopropane) dihydrochloride (AAPH) at a concentration of 153 mM was used. Trolox (0.01 M) was employed as the standard, and fluorescein (55 mM) served as the fluorescent probe, using approximately 25 µL of

concentration??

phosphate buffer (75 mM) at pH 7.4 as the blank. Subsequently, Trolox standard or the

in fidderent concentration?? or how many concentration?

diluted sample in phosphate-buffered saline (PBS) at pH 7.4 was mixed with 250 µL of fluorescein, followed by a 10-minute incubation at 37 °C. An automatic injection of 25 µL of AAPH solution (153 mM) was added to all microplates, and fluorescence was measured every minute for 50 minutes. The final ORAC values were calculated from the area under the curves, expressed as micromoles of Trolox equivalents per gram of dry weight (µmol TE/g dw). This quantitative method provides information about the

antioxidant capacity of the extracts, evaluating their effectiveness in neutralizing peroxyl radicals.

Detection and quantification of polyphenols

A modified methodology by (12) for detecting and quantifying the polyphenol profile. For the detection and quantification of the polyphenol profile, a modified solid-phase extraction (SPE) process was used, employing SiliaPrep C-18 cartridges (17%) 500 mg 3mL, 60 A; previously conditioned with 10 mL of MeOH, 10 mL of ultra-pure water, and then the 8 mL sample; later, it was diluted with 10 mL of MeOH. Subsequently, it was filtered through a membrane with a pore size of 0.22 µm and finally placed in an ultra-high-resolution chromatography (UHPLC), Agilent 1290 II, USA, which is coupled with a Diode Array Detector (DAD). In addition, a Poroshell EC - C18 column (2.1 x 150 mm × 1.9 µm) was used. The chromatographic equipment was set at 30 °C, a flow rate of 0.3 mL min-1, and an injection volume of 1 µL. However, the mobile phase preparation for separation was done using mobile phases such as A (ultra-pure water and 0.1% formic acid) and B (acetonitrile and 0.1% formic acid), which were in gradient mode. The gradient was programmed as follows: 95% A – 5% B for the first 15 min, followed by 60% A – 40% B for the next 18 min, and then a return to 95% A – 5% B over the next 20 min, with a post time of one min. The polyphenol standards considered were those established in Table 1. These standards were mixed and diluted at concentrations ranging from 0.3 to 10 µg/mL for the calibration curves. Additionally, the analyses were performed in triplicate.

Data analysis

The statistical analysis used R Studio version 4.2.1 for the Windows system. The data underwent one-way analysis of variance (ANOVA) after verifying that they met the assumptions of normality through the Shapiro-Wilk test and homogeneity of variances

through Levene's test. Subsequently, a post hoc test was performed, with a significance level (alpha) of 0.05, to determine the presence of significant differences in the data.

Results and discussion

The effect of azithromycin on plant height and dry aboveground biomass

The results obtained regarding plant height and dry aboveground biomass (ear weight, stem weight, and leaf weight) of purple corn (*Zea mays* L.) are presented in Table 1.

Table 1: Plant height and dry aboveground biomass weight according to the different treatments.

**Treatment µ/L** what unit?	**Stem weight** unit=??		**Leaf weight**		is this = stem height?? **Plant height** unit=??		**Ear weight**	
	$\bar{X}$	SD	$\bar{X}$	SD	$\bar{X}$	SD	$\bar{X}$	SD
0	62.64^{ab}	±3.35	36.15^{b}	±4.76	209^{a}	±20.42	7.2^{a}	±0.76
1	71.7^{a}	±8.21	50.31^{a}	±7.18	199^{a}	±21.74	4.8^{b}	±0.52
10	67.16^{a}	±8.66	45.32^{ab}	±7.23	215^{a}	±21	4.2^{bc}	±1.22
100	53.18^{b}	±6.79	37.99^{b}	±5.61	200^{a}	±7.54	3.38^{c}	±0.54

$\bar{X}$: Represents the average. SD: Represents the standard deviation (n= 3). The plant height is expressed in centimeters. The dry weight of the ear, stem weight, and leaf weight are expressed in grams.

Plant height

These findings reveal that *Zea mays* L. was not significantly affected by AZM in plant growth when applied at 1, 10, and 100 µg/L of AZM throughout its vegetative period (Table 1). Additionally, a slight increase of 3% in plant height was observed at a dose of 10 µg/L of AZM compared to the control group (table 1). However, this positive effect disappeared when the concentration was raised to 100 µg/L of AZM, resulting in a 7%

decrease in plant height compared to the 10 µg/L AZM dose, falling below the level of the control group. Therefore, these results suggest that purple corn appears resistant to AZM at low or moderate amounts, but high doses may significantly negatively impact its growth.

Check correct grammar

Plant height was not significantly affected ($P < 0.05$) despite existing scientific literature reporting adverse effects of antibiotic residues on various crops ' sizes (30, 31, 32). Other response mechanisms of this particular species, such as secondary metabolites, could explain this apparent contradiction. Given that this crop is known for its high content of phenolic compounds, primarily flavonoids, and among them, anthocyanins are especially relevant (35). These natural compounds are fundamental to the plant's defense (36). Plants often use the apoplast and other specialized organelles, such as the vacuole, to store these compounds produced through the shikimic acid pathway to respond to external threats (37). These phenolic compounds actively work to counteract the overproduction of reactive oxygen species (ROS) that originates due to the stress generated by the antibiotic present in the plant (38). This stress arises from the overexcitation of chlorophylls and the disruption of the electron transport chain (39). Therefore, there is likely a positive relationship between these natural polyphenols in this species and its ability to cope with environmental stress caused by AZM.

On the other hand, it was observed that the tested concentrations of AZM caused a hormetic response in this crop regarding its height or growth. This could be related to

How about the effect to nitrogen fixing bacterie? Does AZM can affect?Please explain more .

the antibiotic's disruption of nitrogen metabolism in the plant, leading to a significant reduction in nitrate reductase. It is known that nitrate (NO_3^-) is a fundamental nitrogen source for plants (38, 39). For nitrogen metabolism, the enzyme nitrate reductase (NR) converts the nitrate absorbed by plants into nitrite (NO_2^-), while the enzyme nitrite reductase converts nitrite into ammonium (NH_4^+) (42); this situation could have

occurred inside these plants. Similarly, plant cells avoid ammonium toxicity by quickly transforming it into amino acids through the main conversion pathway involving the sequential action of glutamine synthetase and glutamate synthetase (43). In that sense, the alteration in nitrogen metabolism leads to an increase in nitrogen content at lower antibiotic concentrations, while it decreases at higher concentrations (44); this could explain the increase and decrease in plant height observed in the present study. Therefore, it would justify why the species *Zea mays* L. exhibited a hormetic response to the various studied doses of AZM in this study.

Dry biomass

These results show a significant decrease in the weight of the cob at all three applied doses: 1, 10, and 100 µg/L of AZM, compared to the control group. It is important to note that as the AZM dose increases, there is a progressive decrease in cob weight. In this sense, the group exposed to 100 µg/L of AZM shows the lowest cob weight among all the analyzed groups. These findings highlight the negative influence that AZM has on the development of purple corn cobs.

In corn, there are two types of inflorescences: the tassel containing the male flowers at the plant's apex and another containing the female flowers on the side of the plant (45). As for the reduction in tassel weight (male flowers), this weight decreased as the applied doses increased. This could be attributed to increased growth hormones such as gibberellins and auxins; according to (46), there was a decrease in the weight of the corn crop inflorescence when gibberellic acid levels increased in younger spikes. Additionally, it has been reported that auxins reduce the fresh weight of the inflorescence in mature spikes. Likewise, (45, 46) reported that from 12 days before spiking, the levels of indole-3-acetic acid, zeatin riboside, zeatin, and gibberellic acid in the upper and lower spikes increased dramatically and then decreased rapidly. This suggests a strong

relationship between phytohormones and the differentiation of superiority and inferiority in the spikes. In that context, it is possible that AZM, as an abiotic stressor, is contributing to stimulating the increase of these phytohormones in the plant, which could be causing stress in the inflorescence, reducing its dry weight. On the other hand, (49) reported a low presence of polyphenols in this part of the plant, which could have influenced the decrease in the observed inflorescence weight.

Please check. Is it correct reference?

These reveal a significant increase ($P < 0.05$) in stem weight when the plant is exposed to a concentration of 1 µg/L of AZM, compared to the group exposed to 100 µg/L of AZM. This indicates that the applied concentrations of AZM caused a hormetic response regarding stem weight in the species *Zea mays* L. A 14 and 7% increase in stem weight was observed concerning the control group at doses of 1 and 10 µg/L of AZM, respectively. However, at high doses (100 µg/L of AZM), a 15% decrease in stem weight was recorded compared to the control group. These findings highlight the positive and negative influence AZM exerts on stem weight in purple corn. The same phenomenon is observed in leaf weight, as there is a significant difference between the treatment at one and the control group; likewise, once it reaches its peak in leaf weight, it tends to decrease below the control group at the highest dose.

In this study, it was revealed that purple corn could tolerate low concentrations of AZM (1 µg/L of AZM). This is because, at this concentration, an increase in stem weight is observed. This could be attributed to the action of gibberellins, which induce stem elongation. Along with this effect, there is a decrease in stem thickness and a reduction in leaf size (50). This explains why a hormetic response is observed regarding stem and leaf weight. On the other hand, this response could also be related to anthocyanins in the stem and leaves (49).

This highlights the central role of anthocyanins as antioxidants, as they protect the plant from stress by eliminating free radicals produced in AZM-induced oxidative stress. However, it appears less effective at high AZM concentrations (100 µg/L of AZM), as a decrease in leaf and stem weight below the control group is observed. On the other hand, in the study of (51), the absorption of AZM by lettuce plants, which were grown in a sand substrate enriched with biological sludge containing concentrations of AZM considered environmentally relevant, was recorded. In addition, another analysis conducted by (23) indicated that when maize undergoes antibiotic-induced stress, it forms smaller vascular bundles, negatively affecting the transport of photosynthetic substances. Therefore, AZM could be mobilized to different parts of the plant, simultaneously impairing the photosynthesis process in maize, resulting in a decrease in leaf and stem weight and, overall, a reduction in the plant's aerial biomass.

Root length and weight of the root biomass

The results regarding the root length and weight of the root biomass of purple corn (*Zea mays* L.) are presented in Table 2.

Table 2: Root length and weight of dry root biomass according to different ~~treatments~~ concentrations of AZM

Concentration of AZM **Treatment** ug?? **µ/L**	**Root length** add unit in cm		**Dry weight of root biomass** add unit in g	
	$\bar{X}$	SD	$\bar{X}$	SD
0	79.8a	±13.72	21.7a	±11.29
1	85.5a	±13.17	16.4b	±9.13
10	76.5a	±23.18	18.15ab	±19.63
100	77.5a	±13.02	21.7a	±19.53

$\bar{X}$: Represents the average. DE: Represents the standard deviation (n= 3). Root length is expressed in centimeters. The dry weight of root biomass is expressed in grams.

why 0 and 100 ug of AZM cause similar dry weight of root?

The results revealed that *Zea mays* L. did not experience significant effects when exposed to AZM at doses of 1, 10, and 100 μg/L during its vegetative period. However, a reduction in root biomass weight was observed by 24 and 16% at concentrations of 1 and 10 μg/L, respectively, compared to the control group. On the other hand, a slight elongation in root length was observed at a concentration of 1 μg/L compared to the control group. Nevertheless, this positive effect disappeared when the concentration was increased to 10 and 100 μg/L of AZM, resulting in a decrease in root length below the levels of the control group. Therefore, our results suggest that purple corn seems resistant to AZM at low doses, but at doses higher than 10 μg/L, it may negatively impact root biomass and length. These findings indicate that the response of *Zea mays* L. to AZM varies depending on the concentration, and higher doses can negatively affect the plant's root system.

In the different treatments with AZM, there is no significant effect on the root length or root biomass weight of *Zea mays* L. This could be attributed to the glutathione S-transferases (GST) detoxification pathway, where the drug is first catalyzed by cytochrome P450, followed by conjugation mediated by glutathione (GSH) in the cytoplasm, and then transported to the vacuolar compartment for elimination outside the cell (49, 50). Although there was no significant difference between the treatments, a hormetic response has been observed in root length, which may be related to the abscisic acid content in the roots that can serve as a chemical signal in root development and elongation in response to antibiotic stress. Additionally, there is an increase in the antioxidant system, including superoxide dismutase (SOD), peroxidase (POD), and catalase (CAT); this system can be activated by the overproduction of reactive oxygen species (ROS) (54). Therefore, the roots are the main entry pathways for antibiotics into plants (55). The absorption of the antibiotic by plants appears to depend primarily on its

solubility and octanol-water partition coefficients (log P o/w); for AZM, its log P o/w is 3.87–4.02, and it also has water solubility ≥ 0.5 g /L at 25 °C (56) (Ghasemi & Saaidpour, 2007) (57); so, it is within the range (log Kow 1–4) to be easily absorbed by the roots and translocated (58).

Effect of azithromycin on the content of antioxidant compounds

When the concentration of AZM increased from 0 to 10 ug/L, the content of total polyphenols and monomeric anthocyanins increased two times (Figure 1a and 1b). Although no prior research has specifically explored the impact of antibiotics on polyphenol content in purple corn, recent investigations in diverse plant matrices positively affect the production of these secondary metabolites. For example, (12) observed an increase from 1 to 100 ug/L of ciprofloxacin improved by 30% of the total polyphenol content in quinoa grains. This suggests that antibiotics could induce stress conditions within the plant system and promote the production of reactive oxygen species (ROS) or free radicals (55, 56, 57). In response to this stress, plants activate a defense mechanism, synthesizing secondary metabolites (polyphenols) through the shikimic acid pathway (60, 61). These polyphenols neutralize free radicals by donating electrons (hydrogen) (64).

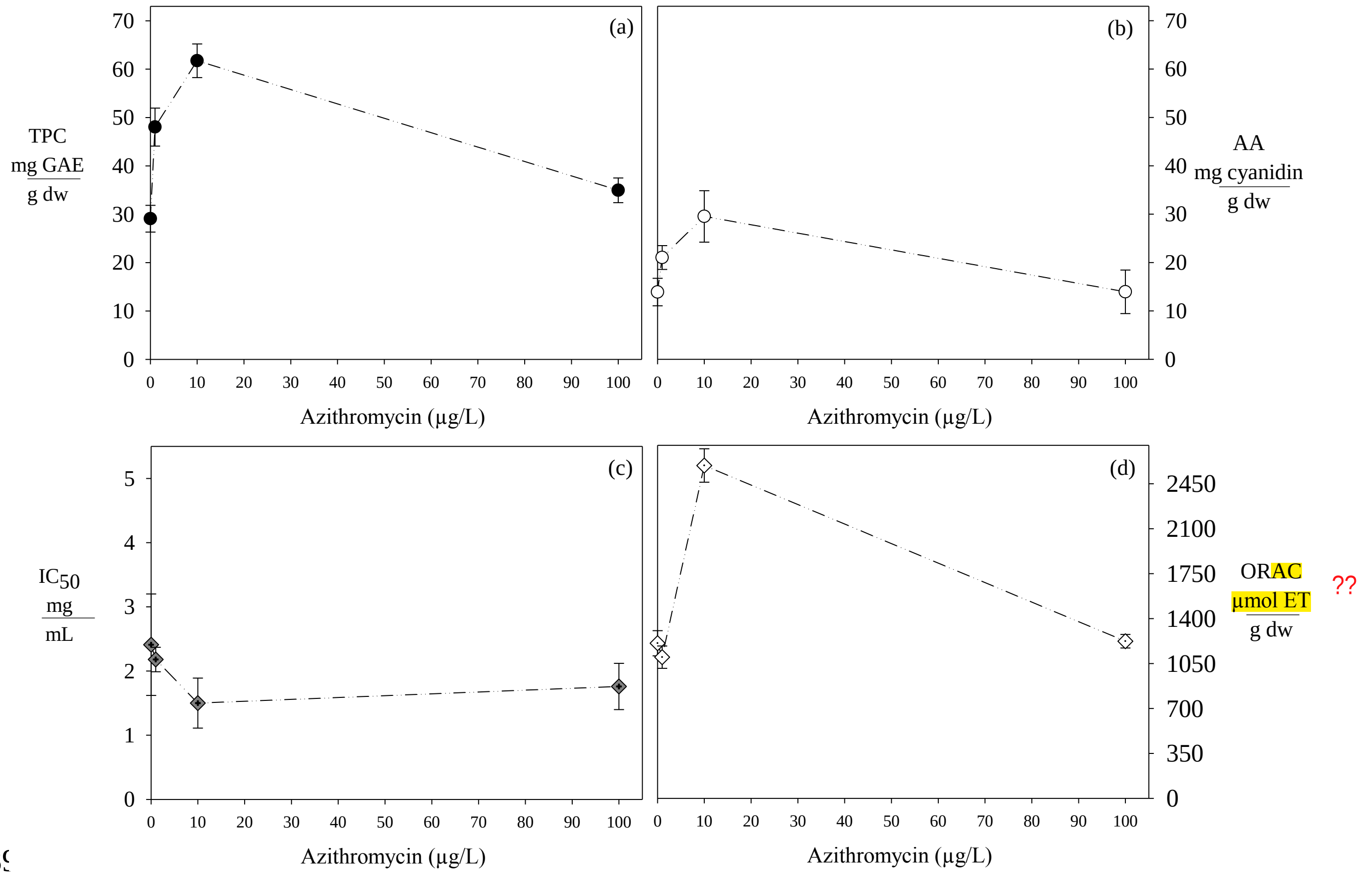

no Figure caption.....?

The total polyphenol content (TPC) is expressed as mg of gallic acid equivalent per gram of dry weight (mg GAE/g dw). The anthocyanin content (AA) is defined as mg of cyanidin-3-glucoside per gram of dry weight. The IC_{50} value is mg of extract per mL of DPPH solution. The ORAC value is the micromoles of Trolox equivalent per gram of dry weight (µmol ET/g dw). Different letters indicate significant differences for each response variable ($p < 0.05$).

On the contrary, when the concentration of AZM increased from 10 to 100 ug/L, total polyphenols and monomeric anthocyanins decreased by 44% and 53%, respectively (Figure 1a and 1b). Two distinct inhibition mechanisms are probably happening in the production of secondary metabolites. According to (65), the presence of elevated

concentrations of antibiotics can generate an excess of reactive oxygen species (ROS); this surplus of ROS can result in the peroxidation of membrane lipids, causing structural damage that, in turn, diminishes the cells' capacity to synthesize polyphenols (66). On the other hand, high concentrations of free radicals could inhibit the activity of critical enzymes like phenylalanine ammonia-lyase and coenzyme A (CoA) ligase in the cytoplasm. Consequently, the production of these metabolites could be reduced (13).

The capacity of polyphenols to neutralize free radicals is known as antioxidant capacity. In this context, the DPPH analysis assesses the effectiveness of polyphenols in neutralizing synthetic radicals, while the ORAC analysis evaluates the ability to reduce biological radicals (67). Thus, conducting both tests to compare the results effectively is advisable. According to our results, ORAC analysis showed the higher the polyphenol content, the higher the antioxidant capacity; conversely, a rise in polyphenol content is associated with a decrease in the IC50 value when it was assessed through the DPPH method (Figure 1c and 1d). In this sense, a high IC50 value indicates that a more significant amount of extract is necessary to inhibit the solution of the DPPH radical.

Effect of azithromycin on specific families of polyphenols

Similar to the behavior reported for total polyphenols (Table 3), the content of distinct polyphenolic families, including flavonols, phenolic acids, and flavonols, are also affected by the presence of AZM (Table 3). For example, when the AZM concentration increased from 0 to 10 µg/L, the content of flavonols, phenolic acids, and flavonoids increased by 57, 28 and 83%, respectively (Table 3). On the contrary, when the concentration of AZM rose from 10 to 100 ug/L, the content of flavonols, phenolic acids, and flavonoids decreased by 17%, 40%, and 42%, respectively (Table 3). As previously discussed, the presence of low antibiotic concentrations promotes the generation of metabolites within the plant system. In contrast, high levels of AZM may potentially

disrupt the integrity of the cell membrane, subsequently diminishing the synthesis of polyphenols. Finally, using 10 ug/L of AZM allowed for obtaining high concentrations of specific polyphenols with 6.71, 2.64, and 3.56 ug/g dw for quercetin, vanillic acid, and epicatechin (Table 3).

Table 3. Chemical characterization of specific families of polyphenols

Description	Treatments							
	0 ug/L		1ug/L		10 ug/L		100 ug/L	
	Mean	SD	Mean	SD	Mean	SD	Mean	SD
**Flavonols** (ug/g dw)								
Rutin	4.68^{a}	±0.83	5.87^{b}	±0.12	6.71^{b}	±1.09	5.75^{b}	±1.07
Quercetin	0.46^{a}	±0.09	0.82^{b}	±0.11	1.37^{c}	±0.17	0.93^{b}	±0.03
Σ	5.14		6.69		8.08		6.68	
**Phenolic acids** (ug/g dw)								
Vanillic	2.06^{a}	±0.22	2.90^{b}	±0.51	2.64^{b}	±0.34	1.57^{c}	±0.17
**Flavanols** (ug/g dw)								
Catechin	0.66^{a}	±0.03	0.69^{a}	±0.08	0.96^{b}	±0.11	0.64^{a}	±0.16
Epicatechin	2.02^{a}	±0.22	4.94^{b}	±0.82	3.56^{c}	±0.21	1.93^{a}	±0.26
Procyanidin B2	0.50^{a}	±0.11	0.76^{b}	±0.09	0.82^{b}	±0.15	0.60^{a}	±0.13
Procyanidin A2	0.75^{a}	±0.12	1.05^{b}	±0.18	1.89^{c}	±0.41	1.06^{b}	±0.15
Σ	3.93		7.44		7.23		4.23	

Conclusion

The presence of AZM in irrigation water at concentrations of 1, 10, and 100 µg/L does not show a statistically significant effect on plant height; however, concerning the weight of the aboveground biomass, the concentration of 1 µg/ L exhibits a statistically significant increase compared to the control. On the other hand, starting from a concentration of 10 up to 100 µg/L, this compound reduces the dry weight of the cob and stem by 41 and 15%, respectively. The root biomass's root length and dry weight do not show a statistically significant effect. However, there is a decrease of 24 and 16% in the dry weight of the root biomass at concentrations of 1 and 10 µg/L, respectively. As for

the concentration of phenolic compounds and total anthocyanins, they double their concentration at a dose of 10 μg/L, and a decrease is observed at a dose of 100 μg/L by 44 and 53%, respectively. The same pattern is observed in antioxidant capacity; regarding the content of flavonols, phenolic acids, and flavonols, they increase by 57, 28, and 83%, respectively, when the concentration of AZM is from 0 to 10 μg/L. However, with an increase from 10 to 100 μg/L, these compounds decrease by 17, 40, and 42%, respectively. These findings highlight the importance of understanding the impact of pharmaceutical contaminants on plant secondary metabolites and their environmental effects.

Acknowledgments

To the Universidad Nacional de Moquegua for financing this Project (Resolución de Comisión Organizadora N° 727–2022-UNAM, the Resolución de Comisión Organizadora N° 0310–2020—UNAM and the Resolución de Comisión Organizadora N° 059–2021—UNAM).

References

1. MIDAGRI. El maíz morado peruano, un producto con alto contenido de antocianina, poderoso antioxidante natural [Internet]. 2021. Available from: https://cdn.www.gob.pe/uploads/document/file/3531000/ El Maíz Morado Peruano.pdf
2. Barba FJ, Rajha HN, Debs E, Abi-Khattar AM, Khabbaz S, Dar BN, et al. Optimization of Polyphenols' Recovery from Purple Corn Cobs Assisted by Infrared Technology and Use of Extracted Anthocyanins as a Natural Colorant in Pickled Turnip. Molecules. 2022;27(16).
3. Cai T, Ge-Zhang S, Song M. Anthocyanins in metabolites of purple corn. Front

Plant Sci. 2023;14(April):1–10.
4. Rodríguez-Miranda J, Carlos-Isidro A, Martínez-Sánchez CE, Herman-Lara E, Torruco-Uco JG, Santiago-Adame R, et al. Phytochemical screening, polyphenols content and antioxidant activity of by-products of two corn variety. Emirates J Food Agric. 2022;35(10):806–14.
5. Gill SS, Tuteja N. Reactive oxygen species and antioxidant machinery in abiotic stress tolerance in crop plants. Plant Physiol Biochem [Internet]. 2010;48(12):909–30. Available from: http://dx.doi.org/10.1016/j.plaphy.2010.08.016
6. Stromsnes K, Lagzdina R, Olaso- gonzalez G, Gimeno- mallench L, Gambini J. Pharmacological properties of polyphenols: Bioavailability, mechanisms of action and biological effects in in vitro studies, animal models and humans. Biomedicines. 2021;9(8).
7. Fan FY, Sang LX, Jiang M, McPhee DJ. Catechins and their therapeutic benefits to inflammatory bowel disease. Molecules. 2017;22(3).
8. Sirerol JA, Rodríguez ML, Mena S, Asensi MA, Estrela JM, Ortega AL. Role of natural stilbenes in the prevention of cancer. Oxid Med Cell Longev. 2016;2016.
9. Daveri E, Cremonini E, Mastaloudis A, Hester SN, Wood SM, Waterhouse AL, et al. Cyanidin and delphinidin modulate inflammation and altered redox signaling improving insulin resistance in high fat-fed mice. Redox Biol. 2018;18(April):16–24.
10. Huaman-Castilla NL, Mariotti-Celis MS, Perez-Correa JR. Polyphenols of Carménère Grapes. Mini Rev Org Chem. 2017;14(3):176–86.
11. Mena P, Calani L, Bruni R, Del Rio D. Bioactivation of High-Molecular-Weight Polyphenols by the Gut Microbiome. Diet-Microbe Interactions in the Gut: Effects on Human Health and Disease. Elsevier Inc.; 2015. 73–101 p.

12. Ramos AQ, Ramos YM, Camaticona NMQ, Tejeda JLR, Quiróz CNC, Maldonado I, et al. Deciphering Ciprofloxacin's Impact on Growth Attributes and Antioxidant Compounds in Pasankalla Quinoa. Agronomy. 2023;13(7):1738.
13. Wang J, Xu J, Gong X, Yang M, Zhang C, Li M. Biosynthesis, chemistry, and pharmacology of polyphenols from Chinese Salvia species: A review. Molecules. 2019;24(1):1–23.
14. Zagoskina N V., Zubova MY, Nechaeva TL, Kazantseva V V., Goncharuk EA, Katanskaya VM, et al. Polyphenols in Plants: Structure, Biosynthesis, Abiotic Stress Regulation, and Practical Applications (Review). Int J Mol Sci. 2023;24(18):13874.
15. Marano RBM, Zolti A, Jurkevitch E, Cytryn E. Antibiotic resistance and class 1 integron gene dynamics along effluent, reclaimed wastewater irrigated soil, crop continua: elucidating potential risks and ecological constraints. Water Res [Internet]. 2019;164:114906. Available from: https://doi.org/10.1016/j.watres.2019.114906
16. Oharisi O oke L, Ncube S, Nyoni H, Madikizela ML, Olowoyo OJ, Maseko BR. Occurrence and prevalence of antibiotics in wastewater treatment plants and effluent receiving rivers in South Africa using UHPLC-MS determination. J Environ Manage [Internet]. 2023;345(July):118621. Available from: https://doi.org/10.1016/j.jenvman.2023.118621
17. Nieto-Juárez JI, Torres-Palma RA, Botero-Coy AM, Hernández F. Pharmaceuticals and environmental risk assessment in municipal wastewater treatment plants and rivers from Peru. Environ Int. 2021;155(May):106674.
18. Mishra S, Kumar R, Kumar M. Use of treated sewage or wastewater as an irrigation water for agricultural purposes- Environmental, health, and economic impacts.

Total Environ Res Themes. 2023;6(March):100051.

19. Ofori S, Puškáčová A, Růžičková I, Wanner J. Treated wastewater reuse for irrigation: Pros and cons. Sci Total Environ. 2021;760.

20. Gudda F, Odinga ES, Tang L, Waigi MG, Wang J, Abdalmegeed D, et al. Tetracyclines uptake from irrigation water by vegetables: Accumulation and antimicrobial resistance risks. Environ Pollut [Internet]. 2023;338(September):122696. Available from: https://doi.org/10.1016/j.envpol.2023.122696

21. Ben Mordechay E, Mordehay V, Tarchitzky J, Chefetz B. Pharmaceuticals in edible crops irrigated with reclaimed wastewater: Evidence from a large survey in Israel. J Hazard Mater [Internet]. 2021;416:126184. Available from: https://doi.org/10.1016/j.jhazmat.2021.126184

22. Marques RZ, Wistuba N, Brito JCM, Bernardoni V, Rocha DC, Gomes MP. Crop irrigation (soybean, bean, and corn) with enrofloxacin-contaminated water leads to yield reductions and antibiotic accumulation. Ecotoxicol Environ Saf. 2021;216(February):112193.

23. Gomes MP, Rocha DC, Moreira de Brito JC, Tavares DS, Marques RZ, Soffiatti P, et al. Emerging contaminants in water used for maize irrigation: Economic and food safety losses associated with ciprofloxacin and glyphosate. Ecotoxicol Environ Saf [Internet]. 2020;196(March):110549. Available from: https://doi.org/10.1016/j.ecoenv.2020.110549

24. González-Muñoz A, Quesille-Villalobos AM, Fuentealba C, Shetty K, Gálvez Ranilla L. Potential of Chilean native corn (Zea mays L.) accessions as natural sources of phenolic antioxidants and in vitro bioactivity for hyperglycemia and hypertension management. J Agric Food Chem. 2013;61(46):10995–1007.

25. Chirinos R, Campos D, Costa N, Arbizu C, Pedreschi R, Larondelle Y. Phenolic profiles of andean mashua (Tropaeolum tuberosum Ruíz & Pavón) tubers: Identification by HPLC-DAD and evaluation of their antioxidant activity. Food Chem. 2008;106(3):1285–98.

26. Singleton VL, Rossi JAJ. Colorimetry to total phenolics with phosphomolybdic acid reagents. Am J Enol Vitic [Internet]. 1965;16(48):144–58. Available from: http://garfield.library.upenn.edu/classics1985/A1985AUG6900001.pdf

27. Huaman C. Caracterización de los compuestos fenolicos del germoplasma de maíz (Zea mays L.) nativo de la región Arequipa, Perú. Universidad Nacional Agraria La Molina; 2017. p. 17–24.

28. Hosseinian FS, Li W, Beta T. Measurement of anthocyanins and other phytochemicals in purple wheat. Food Chem. 2008;109(4):916–24.

29. Shao Y, Xu F, Sun X, Bao J, Beta T. Identification and quantification of phenolic acids and anthocyanins as antioxidants in bran, embryo and endosperm of white, red and black rice kernels (Oryza sativa L.). J Cereal Sci [Internet]. 2014;59(2):211–8. Available from: http://dx.doi.org/10.1016/j.jcs.2014.01.004

30. Brand-Williams W, Cuvelier ME, Berset C. Use of a free radical method to evaluate antioxidant activity. LWT - Food Sci Technol. 1995;28(1):25–30.

31. Gálvez Ranilla L, Christopher A, Sarkar D, Shetty K, Chirinos R, Campos D. Phenolic Composition and Evaluation of the Antimicrobial Activity of Free and Bound Phenolic Fractions from a Peruvian Purple Corn (Zea mays L.) Accession. J Food Sci. 2017;82(12):2968–76.

32. Khan KY, Ali B, Zhang S, Stoffella PJ, Yuan S, Xia Q, et al. Effects of antibiotics stress on growth variables, ultrastructure, and metabolite pattern of Brassica rapa ssp. chinensis. Sci Total Environ [Internet]. 2021;778:146333. Available from:

https://doi.org/10.1016/j.scitotenv.2021.146333

33. Vilca FZ, Loayza ODV, Ponce TEL, Junqueira LV, Galarza NC, Torres NH, et al. Presence of enrofloxacin residues in soil and its effect on carbon fixation, number of nodules, and root length of alfalfa (Medicago sativa). J Hazard Mater Adv [Internet]. 2022;7(April):100100. Available from: https://doi.org/10.1016/j.hazadv.2022.100100

34. Chen H, Jin J, Hu S, Shen L, Zhang P, Li Z, et al. Metabolomics and proteomics reveal the toxicological mechanisms of florfenicol stress on wheat (Triticum aestivum L.) seedlings. J Hazard Mater. 2023;443(September 2022).

35. Suriano S, Balconi C, Valoti P, Redaelli R. Comparison of total polyphenols, profile anthocyanins, color analysis, carotenoids and tocols in pigmented maize. Lwt [Internet]. 2021;144(September 2020):111257. Available from: https://doi.org/10.1016/j.lwt.2021.111257

36. Adhikary S, Dasgupta N. Role of secondary metabolites in plant homeostasis during biotic stress. Biocatal Agric Biotechnol [Internet]. 2023;50(May):102712. Available from: https://doi.org/10.1016/j.bcab.2023.102712

37. Anjali, Kumar S, Korra T, Thakur R, Arutselvan R, Kashyap AS, et al. Role of plant secondary metabolites in defence and transcriptional regulation in response to biotic stress. Plant Stress [Internet]. 2023;8(March):100154. Available from: https://doi.org/10.1016/j.stress.2023.100154

38. Mittler R. ROS Are Good. Trends Plant Sci. 2017;22(1):11–9.

39. Nunes B, Veiga V, Frankenbach S, Serôdio J, Pinto G. Evaluation of physiological changes induced by the fluoroquinolone antibiotic ciprofloxacin in the freshwater macrophyte species Lemna minor and Lemna gibba. Environ Toxicol Pharmacol [Internet]. 2019;72(August):103242. Available from:

https://doi.org/10.1016/j.etap.2019.103242

40. Sun Y, Hou M, Mur LAJ, Yang Y, Zhang T, Xu X, et al. Nitrogen drives plant growth to the detriment of leaf sugar and steviol glycosides metabolisms in Stevia (Stevia rebaudiana Bertoni). Plant Physiol Biochem [Internet]. 2019;141(April):240–9. Available from: https://doi.org/10.1016/j.plaphy.2019.06.008

41. An T, Wu Y, Xu B, Zhang S, Deng X, Zhang Y, et al. Nitrogen supply improved plant growth and Cd translocation in maize at the silking and physiological maturity under moderate Cd stress. Ecotoxicol Environ Saf [Internet]. 2022;230:113137. Available from: https://doi.org/10.1016/j.ecoenv.2021.113137

42. Oliveira HC, Freschi L, Sodek L. Nitrogen metabolism and translocation in soybean plants subjected to root oxygen deficiency. Plant Physiol Biochem [Internet]. 2013;66:141–9. Available from: http://dx.doi.org/10.1016/j.plaphy.2013.02.015

43. Zhou Q, Gao J, Zhang R, Zhang R. Ammonia stress on nitrogen metabolism in tolerant aquatic plant—Myriophyllum aquaticum. Ecotoxicol Environ Saf [Internet]. 2017;143(May):102–10. Available from: https://doi.org/10.1016/j.ecoenv.2017.04.016

44. Fiaz M, Ahmed I, Hassan SMU, Niazi AK, Khokhar MF, Zeshan, et al. Antibiotics induced changes in nitrogen metabolism and antioxidative enzymes in mung bean (Vigna radiata). Sci Total Environ [Internet]. 2023;873(November 2022):162449. Available from: https://doi.org/10.1016/j.scitotenv.2023.162449

45. Yao H, Skirpan A, Wardell B, Matthes MS, Best NB, McCubbin T, et al. The barren stalk2 Gene Is Required for Axillary Meristem Development in Maize. Mol Plant [Internet]. 2019;12(3):374–89. Available from:

https://doi.org/10.1016/j.molp.2018.12.024

46. BOMMINENI VR, GREYSON R. Effect of gibberelic acid and IIA on growth and differentiation of cultured ear inflorescences of maize (Zea mays). 1990;68:239–47.

47. DU K, ZHAO W qing, ZHOU Z guo, SHAO J jing, HU W, KONG L jie, et al. Hormonal changes play important roles in the key period of superior and inferior earshoot differentiation in maize. J Integr Agric. 2021;20(12):3143–55.

48. Zhao M, Li J, Zhou S, Li K, Niu L, Zhao L, et al. Analysis of the effects of sulfamethoxazole on the secondary metabolites and antioxidants in oilseed rape (Brassica napus L.) and the underlying mechanisms. Sci Total Environ. 2023;902(May).

49. Paulsmeyer MN, Vermillion KE, Juvik JA. Assessing the diversity of anthocyanin composition in various tissues of purple corn (Zea mays L.). Phytochemistry [Internet]. 2022;201(May):113263. Available from: https://doi.org/10.1016/j.phytochem.2022.113263

50. Guzmán Y, Pugliese B, González C V., Travaglia C, Bottini R, Berli F. Spray with plant growth regulators at full bloom may improve quality for storage of "Superior Seedless" table grapes by modifying the vascular system of the bunch. Postharvest Biol Technol. 2021;176(August 2020).

51. Sidhu H, O'Connor G, Kruse J. Plant toxicity and accumulation of biosolids-borne ciprofloxacin and azithromycin. Sci Total Environ [Internet]. 2019;648:1219–26. Available from: https://doi.org/10.1016/j.scitotenv.2018.08.218

52. Sun C, Dudley S, McGinnis M, Trumble J, Gan J. Acetaminophen detoxification in cucumber plants via induction of glutathione S-transferases. Sci Total Environ [Internet]. 2019;649:431–9. Available from:

https://doi.org/10.1016/j.scitotenv.2018.08.346

53. Marques RZ, Wistuba N, Brito JCM, Bernardoni V, Rocha DC, Gomes MP. Crop irrigation (soybean, bean, and corn) with enrofloxacin-contaminated water leads to yield reductions and antibiotic accumulation. Ecotoxicol Environ Saf. 2021;216(January).

54. Li L, Li T, Liu Y, Li L, Huang X, Xie J. Effects of antibiotics stress on root development, seedling growth, antioxidant status and abscisic acid level in wheat (Triticum aestivum L.). Ecotoxicol Environ Saf. 2023;252(June 2022).

55. Rocha DC, da Silva Rocha C, Tavares DS, de Morais Calado SL, Gomes MP. Veterinary antibiotics and plant physiology: An overview. Sci Total Environ [Internet]. 2021;767:144902. Available from: https://doi.org/10.1016/j.scitotenv.2020.144902

56. Ghasemi J, Saaidpour S. Quantitative structure-property relationship study of n-octanol-water partition coefficients of some of diverse drugs using multiple linear regression. Anal Chim Acta. 2007;604(2):99–106.

57. González-Pleiter M, Pedrouzo-Rodríguez A, Verdú I, Leganés F, Marco E, Rosal R, et al. Microplastics as vectors of the antibiotics azithromycin and clarithromycin: Effects towards freshwater microalgae. Chemosphere. 2021;268.

58. Bagheri M, Al-jabery K, Wunsch DC, Burken JG. A deeper look at plant uptake of environmental contaminants using intelligent approaches. Sci Total Environ [Internet]. 2019;651:561–9. Available from: https://doi.org/10.1016/j.scitotenv.2018.09.048

59. Alscher RG, Donahue JL, Cramer CL. Reactive oxygen species and antioxidants: Relationships in green cells. Physiol Plant. 1997;100(2):224–33.

60. Tiwari S. Reactive oxygen species and antioxidants: A continuous scuffle within

the cell. React Oxyg Species Plants Boon Or Bane - Revisiting Role ROS. 2017;187–203.

61. Zhang Y, Xu M, Liu X, Wang M, Zhao J, Li S, et al. Regulation of biochar mediated catalytic degradation of quinolone antibiotics: Important role of environmentally persistent free radicals. Bioresour Technol. 2021;326(January):124780.

62. Kumar S, Abedin MM, Kumar A, Das S. Plant Phenolics in Sustainable Agriculture: Volume 1. In: Role of Phenolic Compounds in Plant-Defensive Mechanisms. 2020. p. 517.

63. Šamec D, Karalija E, Šola I, Vujčić Bok V, Salopek-Sondi B. The role of polyphenols in abiotic stress response: The influence of molecular structure. Plants. 2021;10(1):1–24.

64. Nimse SB, Pal D. Free radicals, natural antioxidants, and their reaction mechanisms. RSC Adv. 2015;5(35):27986–8006.

65. Jin J, Xu L, Zhang S, Jin MK, Zhang P, Shen L, et al. Oxidative response of rice (Oryza sativa L.) seedlings to quinolone antibiotics and its correlation with phyllosphere microbes and antibiotic resistance genes. Sci Total Environ [Internet]. 2023;867(January):161544. Available from: https://doi.org/10.1016/j.scitotenv.2023.161544

66. Sharma P, Jha AB, Dubey RS, Pessarakli M. Reactive Oxygen Species, Oxidative Damage, and Antioxidative Defense Mechanism in Plants under Stressful Conditions. J Bot. 2012;2012:1–26.

67. Huamán-Castilla NL, Copa-Chipana C, Mamani-Apaza LO, Luque-Vilca OM, Campos-Quiróz CN, Zirena-Vilca F, et al. Selective Recovery of Polyphenols from Discarded Blueberries (Vaccinium corymbosum L.) Using Hot Pressurized Liquid

Extraction Combined with Isopropanol as an Environmentally Friendly Solvent. Foods. 2023;12(19):1–11.
