# Supplementary material for: Divergent effects of azithromycin on purple corn (Zea mays L.) cultivation: Impact on biomass and antioxidant compounds
Source: PLoS One. 2024 Aug 22;19(8):e0307548. doi: 10.1371/journal.pone.0307548 (PMC11340972; doi:10.1371/journal.pone.0307548)
Supplement: Attachment — Submitted filename: Response to Reviewers[F].pdf [file pone.0307548.s003.pdf]

Ilo, May 29th, 2024

Mr.

Editor of the Plos One Journal

Present
Subject: Raising of observations

In response to the request for reference and comments made by the referees to the manuscript "**Divergent effects of Azithromycin on purple corn (*Zea mays L.*) cultivation: Impact on biomass and antioxidant compounds**" In this regard, at the beginning, we thank the referees for their timely comments on the manuscript, which contributed significantly to its improvement. At the same time, we indicate that the comments have been corrected, also here we respond to the comments made by reviewers:

a) Reviewer #1

1) The manuscript describes the AZM's effect on corn's growth and secondary metabolism. The authors performed experiments with four different concentrations of AZM with four biological replications each. It seems like the differences observed in this study is difficult to say that it is only caused by the AZM concentration. In such a big size pot experiment, it is hard to be consistent among replications and moreover, such a long term experiment would have more chance to be screwed with unwanted effects. To verify the changes were caused by AZM treatment It should first be confirmed that AZM is transformed into plant tissues. How much is remained in soil, drain water? Is it caused by microbiome in the soil?

Answer:

We greatly appreciate your insightful comments and observations. We acknowledge that it is indeed challenging to categorically state that the differences observed in our study are solely due to the concentration of azithromycin (AZM). However, prior to setting up the experiment, we took several precautions to minimize external variabilities that could potentially affect our results. For instance, we prepared the substrate uniformly and distributed it evenly across the pots to ensure consistency among the experimental units, varying only the concentrations of AZM in the irrigation. The irrigation water used was distilled water to ensure that only the AZM concentrations were present in this water.

Regarding the transformation and presence of AZM in plant tissues, we based our methodology on previous studies that have demonstrated AZM's ability to enter and bioaccumulate in plant tissues due to its physicochemical properties. For example, Sidhu, O'Connor, & Kruse (2019) documented the translocation of azithromycin in plant tissues, which supported our decision not to perform additional quantification of AZM in the tissues in this study. Similarly, Almeida et al. (2021) did not directly quantify azithromycin in exposed algae, focusing instead on biological response parameters such as growth rate and pigment content.

The bioaccumulation of AZM in plants is correlated with its high aromaticity and higher log Dow value, indicating a high potential for bioaccumulation. The octanol-water distribution ratio (Dow) values of organic compounds are often well correlated with absorption in organic tissues (ECETOC, 2013). Consequently, we assumed that the residual concentration of AZM in the soil and drainage water would be minimal,

given its preferential absorption by the plant. Additionally, studies such as those by Sidhu, O'Connor, Ogram, et al. (2019) have shown minimal toxicity of AZM at concentrations up to 0.06 mg kg-1 for soil microbial activity, suggesting that in our study, with significantly lower concentrations, the soil microbiome would not have significantly interfered with AZM absorption by purple corn.

To corroborate that the soil microbiome did not affect the absorption of azithromycin, we compared the phenolic compound results in the control (without azithromycin) with the values reported in the literature (Shahidi & Ambigaipalan, 2015). We observed that the concentrations of polyphenols increased with AZM concentrations of 1 and 10 μg L-1, suggesting the translocation of AZM within the plant.

We thank you once again for your valuable observations. While it would be ideal to conduct direct measurements of AZM in plant tissues in future studies, the available evidence and the precautions we have taken support the conclusions expressed in the present study.

Almeida, A. C., Gomes, T., Lomba, J. A. B., & Lillicrap, A. (2021). Specific toxicity of azithromycin to the freshwater microalga Raphidocelis subcapitata. *Ecotoxicology and Environmental Safety*, *222*. https://doi.org/10.1016/j.ecoenv.2021.112553

ECETOC, (European Centre for Ecotoxicology and Toxicology of Chemicals). (2013). *ECETOC Technical Report No. 117 - Understanding the Relationship between Extraction Technique and Bioavailability*. *117*.

Shahidi, F., & Ambigaipalan, P. (2015). Phenolics and polyphenolics in foods, beverages and spices: Antioxidant activity and health effects - A review. *Journal of Functional Foods*, *18*, 820–897. https://doi.org/10.1016/j.jff.2015.06.018

Sidhu, H., O'Connor, G., & Kruse, J. (2019). Plant toxicity and accumulation of biosolids-borne ciprofloxacin and azithromycin. *Science of the Total Environment*, *648*, 1219–1226. https://doi.org/10.1016/j.scitotenv.2018.08.218

Sidhu, H., O'Connor, G., Ogram, A., & Kumar, K. (2019). Bioavailability of biosolids-borne ciprofloxacin and azithromycin to terrestrial organisms: Microbial toxicity and earthworm responses. *Science of the Total Environment*, *650*, 18–26. https://doi.org/10.1016/j.scitotenv.2018.09.004

2) The following questions should also be addressed. L97 Which seeds? Plant materials should be specified. eg, cultivar or inbreds

Answer:

Additional information was added in lines L102 to L104 regarding the seeds used in the present study.

3) L108 Be consistent in displaying units and spacing between numbers and units throughout the manuscript, eg, μg or ug in L422, L434, Table 3 treatments, etc.

Answer:

The units in Table 3 and the spacing between numbers and units throughout the article's manuscript were modified.

4) L125 Which samples? The plants were divided into roots, leaves, and cobs in L118. Did you use whole cob sample?

Answer:

Line L137 specifies the sample. Lines L127 to L131 added more information about using the samples and the division process. Furthermore, regarding the cob, it was specified that only the core was used in lines L131 to L135.

5) L234. Table 1. Ear weight was signicantly reduced as the AZM concentration was increased. What about the 100-kernel weight?

Answer:

The term "Ear weight" was changed to "Tassel weight" in Table 1, as grains are not found in the tassel. The grains were not weighed.

b) Reviewer #2

1) Please do language check.

Answer:

The language and spelling of the entire article were checked.

2) Please check some reference at no.49 in text, wether it is correct position or not.

Answer:

Reference No. 49 was checked and the content of the citation was clarified in lines L324 to L327.

3) Introduction : Please explain how AZM will be the residue in water.

Answer:

More information was added in lines L70 to L80 about how AZM turns into residue in water.

4) Line 120: plate height means stem length ?? Please clarify the word and check consistency.

Answer:

The manuscript's coherence was checked. Additionally, it was clarified that only the term "stem length" will be used throughout the manuscript.

5) Explain how to separate stem and how to measure or give the picture of each part of cob to show your measurement (If possible).

Answer:

More information about the measurement process was added in lines L127 to L130.

6) Line 131: %/% =??? Please clarify.

Answer:

The (%/%/) was changed to (v/v) in line L143

7) Line 135: How to be sure that water also removed? , Since you did not freeze dry?

Answer:

Additional information about the mechanism for water evaporation was added in lines L147 to L149.

8) Line 136: Clarify how to prepare sample to final volume 10 ml or you add 10 ml of 60% acetone which the final volume of each sample will be the same??

Answer:

Lines L150 to L152 detail the process of reconstituting the phenolic compounds in 10 mL of 60% acetone.

9) Line 141 ; Please rewrite this sentence with reference 26 and 27

Answer:

The sentence in lines L156 was rewritten.

10) Line 169: Equation (2) and what are gbh and gbs??

Answer:

The units in line L184 were modified, and their meanings were added in lines L190 to L191.

$$TAC\ \left(\frac{mg\ EC3G}{L}\right) = \frac{A \times MW\ \times FD \times 1000}{eL} \times \frac{V}{g\ fw} \times \frac{g\ fw}{g\ dw}$$

11) Line 179: How to prepare the solution in different concentrations of extracts?

Answer:

Additional information was added about the preparation of the solution in different concentrations of extracts in lines L194 to L198.

12) Line 195-195: Explain the concentration of Trolox and the sample that were used to find IC50.

Answer:

The concentration of Trolox and the sample used were added in lines L214 to L218.

13) Line 207 : What is 17% ??.

Answer:

This refers to the percentage of C18 contained in the extraction cartridge.

14) Line 210: This membrane means the syringe filter ?

Answer:

The sentence was modified to explain that it was filtered through a syringe filter with a pore size of 0.22 µm in line L233.

15) Line 232 and Table 1: Plant height = stem length?

Answer:

It was clarified that only the term "stem length" will be used throughout the manuscript in Table 1, L257

16) Table 1: Please correct and write the unit for weight, height and concentration of AZM

Answer:

The units for weight, height, and AZM concentration were corrected in Table 1.

17) Line 265: How about the effect to nitrogen fixing bacteria? Does AZM can affect? Please explain more

Answer:

Additional information about the effect of AZM on nitrogen-fixing bacteria was added in lines L285 to L291.

18) Table2: Add unit and correct the letter which indicated significant difference (a,b)

Answer:

The unit was added and the letter indicating a significant difference was corrected in Table 2

19) Table 2: Why do 0 and 100 ug of AZM cause similar dry weight of root?

Answer:

When using only one unit, the results are similar. However, when working with 2 decimal places, a variation in the decimal figures is noticeable. For this reason, it was decided to use 2 decimal places in Table 2.

20) Line 390 : Figure 1 has no caption

Answer:

The title was added to Figure 1.

21) Line 395: umole ET/g =?? Is it correct?

Answer:

In line L412, the units (µmol ET/g dw) were corrected to (µmol TE/g dw).

22) Table 3: Treatments means treatment with ? should give more details

Answer:

The treatments are detailed in Table 3.

23) References: Check and correct the format.

Answer:

The format was checked and corrected.

c) Reviewer #3

Dear Authors, after a reviewing process I recommend your manuscript to be published at present form.

Firmado digitalmente por ZIRENA VILCA Franz FAU 20449347448 soft
Motivo: Soy el autor del documento
Fecha: 29.05.2024 18:56:38 -05:00

Franz Zirena Vilca

Professor
